# Efficacy of Hydrophobic Layer on Sealing Ability of Dentin Adhesive Systems in Class V Composite Resin Restorations

**DOI:** 10.5681/joddd.2011.002

**Published:** 2011-03-18

**Authors:** Fatemeh Maleknejad, Saied Mostafa Moazzami, Elham Baradaran Nasseri, Ehsan Baradaran Nasseri

**Affiliations:** ^1^ Associate Professor, Department of Operative Dentistry, School of Dentistry, Mashhad University of Medical Sciences, Mashhad, Iran; ^2^ Dentist, Private Practice, Mashhad, Iran; ^3^ Postgraguate Student, Department of Operative Dentistry, Dental School, Kerman University of Medical Sciences, Kerman, Iran

**Keywords:** Dentin adhesive system, hydrophobic layer, microleakage

## Abstract

**Background and aims:**

Adhesive permeability is hindered by application of an additional layer of hydrophobic resin, which increases its concentration within the hydrophilic layer, reduces its affinity to water, and enhances its physical prop-erties. The aim of the present study was to evaluate the effect of a hydrophobic layer on the microleakage of class V composite restorations using different adhesives.

**Materials and methods:**

The adhesives including total-etch Scotchbond MP and Single Bond, and the self-etch Clearfil SE Bond and Clearfil S3 Bond were applied to 80 class V cavities in vitro on the buccal surface in CEJ and then were fol-lowed by hydrophobic resin (Margin Bond) in half of the cavities in each group (n=10). After restoration with microhybrid composite, Z100 and immersion in fuchsine, the degree of microleakage was assessed. Data were analyzed using the Krus-kal-Wallis, Man-Whitney, and Wilcoxon tests.

**Results:**

The hydrophobic layer significantly reduced the microleakage of Clearfil SE Bond and Clearfil S3 Bond only in dentin (p<0.05). There was no significant difference between enamel and dentinal margins in Clearfil S3 Bond, Clearfil SE Bond plus Margin Bond, and Clearfil S3 Bond plus Margin Bond (p>0.05).

**Conclusion:**

Within the limitation of this study, only Clearfil S3 Bond could demonstrate the identical values of microle-akage in enamel and dentinal margins.

## Introduction

Dentin adhesives are currently available as single-step, two-step, and three-step systems. Two-step systems are either available as self-priming adhesives that need a separate etching step or as self-etching primers that require an additional bonding step. The three-step procedures have been combined into a one-step application in the recently introduced all-in-one adhesives.^1^

When the primer and the adhesive have joined into one bottle, it requires more hydrophilic formulations of solvents and monomers.^2^ These adhesives have hydrophilic polymers that are permeable to water movement after polymerization.^3^ Such evidence has led to the fact that current self-etching adhesives would be more hydrophilic and could perform better if it is followed by a hydrophobic resin layer.^4^ Brackett et al^5^ concluded that the addition of a more hydrophobic resin layer following the application of the three self-etching adhesive systems produced significantly higher bond strength. Pushpa & Suresh^6^ applied one-step self-etch adhesives in class V cavity preparations and demonstrated that the sealing ability of one-step adhesives could be improved by the application of more hydrophobic resin layers. The adhesive layer may help to preserve the integrity of hybridized dentin, protecting it from polymerization shrinkage stress and acting as a stress absorbing layer.^7^

In order to create a relatively thick intermediate layer with low elastic modulus between dentin and composite, one option is to apply a second adhesive layer,^8^ as the adhesive layer thickness is not enough to act as a stress absorbing layer and thicker layers would absorb greater stress.^9^ Silva et al^10^ demonstrated that applying an additional layer of solvent-free adhesive systems increased adhesive thickness and preserved the integrity of restoration by acting as a stress absorbing layer. There are some previous studies about the application of flowable composite resin lining as a stress absorbing layer, but with controversial results.^9,11,12^

This in vitro study compared adhesive systems to determine whether the addition of hydrophobic resin layers to the self-etching system would decrease the microleakage of composite restored class V cavities. The null hypotheses of this study regarding the degree of microleakage were as follows: (1) there are no significant differences among enamel margins; (2) there are no significant differences among dentinal margins; (3) there are no significant differences between two similar groups (with and without a hydrophobic layer); and (4) there are no significant differences between enamel and dentinal margins of each restoration.

## Materials and Methods

Eighty freshly caries-free human premolar teeth were extracted out of the orthodontic reason and stored in distilled water for up to 1 month. After cleaning with a rubber cup and slurry of pumice, class V cavity preparation was prepared on the buccal surface of each tooth using a FG coarse diamond bur (8351009, SS White, UK) in a high speed handpiece under water cooling. Cavities (4 mm length, 2.5 mm width and 1.5 mm depth) were prepared in the cementoenamel junction. The dimensions of each cavity were measured with a digital caliper (Mitutoyo, USA). The specimens were randomly assigned to eight groups (n=10) as follows:

Group 1 (Etch and rinse 3-step system): Scotchbond Etchant (35% phosphoric acid, pH= 0.03-0.05, 3M ESPE, St. Paul, MN, USA) was applied for 15 seconds then rinsed with water for 30 seconds. Then Scotchbond Multi-Purpose Plus (3M ESPE, St. Paul, MN, USA) was applied according to manufacturers’ instructions.

Group 2 (Etch and rinse 2-step system): Scotchbond Etchant (35% phosphoric acid, pH= 0.03-0.05, 3M ESPE, St. Paul, MN, USA) was applied for 15 seconds then rinsed with water for 30 seconds. Then Single Bond (3M ESPE, St. Paul, MN, USA) was applied according to manufacturers’ instructions.

Group 3 (Mild self-etch adhesive system): Clearfil SE Bond (Kuraray Medical Inc., Okayama, Japan, pH= 2), Mode of application: Apply primer for 20 seconds. Mild air stream. Apply Bond. Gentle air stream. Light cure for 10 seconds.

Group 4 (All-in-one self-etch adhesive): Clearfil S3 Bond (Kuraray Medical Inc., Okayama, Japan, pH= 2.7). Mode of application: Apply adhesive for 20 seconds. Air-dry with high- pressure for 10 seconds. Light cure for 10 seconds.

Groups 5 through 8 were assigned to the same adhesives respectively except for an additional layer of a more hydrophobic unfilled resin; Margin Bond (Coltene Whaledent, USA) was applied, and air thinned and light cured before the addition of the resin composite restorative material. Other products were applied according to their manufacturers’ instructions.

The cavities were filled with the microhybrid composite resin Z100 (3M ESPE, USA) in two successive oblique layers. Each increment was polymerized using Astralis 7 with intensity of 700 mW/cm^2^ (Ivoclar,Vivadent, Schaan/Liechtenstein, Switzerland) for 40 seconds. The restorations were finished with diamond burs and polished with disks (KerrHawe, Bioggio, Switzerland).

The restored teeth were left overnight in distilled water at room temperature and thermocycled (500 cycles, 5°C ± 2°C to 55°C ± 2°C, 30 seconds dwell time)^13^ to evaluate the microleakage of the restoration over time rather than immediately after placement.^14^The specimens were prepared for microleakage evaluation by coating the entire tooth with one application of nail varnish except for 1 mm around the restoration margin. Specimens were then immersed in a solution of 0.5% basic fuchsine dye for 24 h. Specimens were embedded in phenolic rings with epoxy resin and were sectioned longitudinally in a buccolingual direction with a low speed water-cooled diamond saw.

The staining along both enamel and dentinal restoration interfaces was recorded according to the following criteria: 0: no dye penetration, 1: dye penetration at the interface to 1/2 depth of the cavity wall, 2: dye penetration to the full depth of the cavity wall but not including the axial wall, 3: dye penetration to and along the axial wall. For evaluation of dye penetration, both sides of each section was viewed by stereomicroscope (×30 magnification)

The enamel and dentinal scores in the experimental groups were compared with the Kruskal-Wallis and Mann-Whitney nonparametric tests. Combined enamel and dentinal mean scores within each restoration were compared with the Wilcoxon matched-pairs signed rank test (P<0.05). All analyses were performed with the SPSS software, version 16.0.

## Results

**Figure 1:**
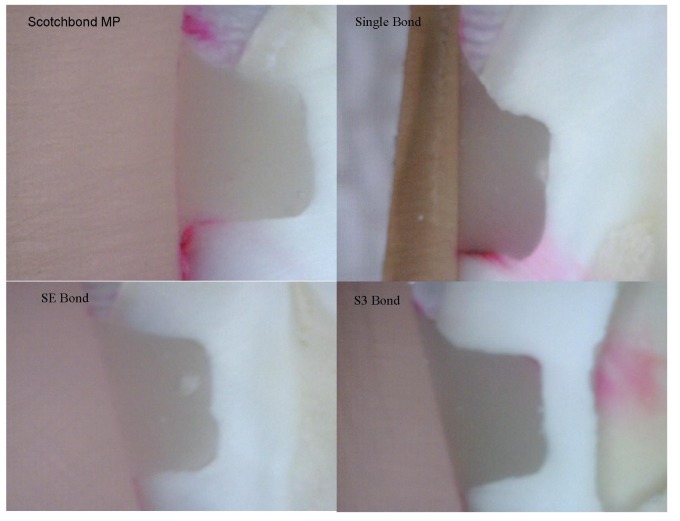
Representation of dye penetration in groups included with hydrophobic layer.

**Figure 2:**
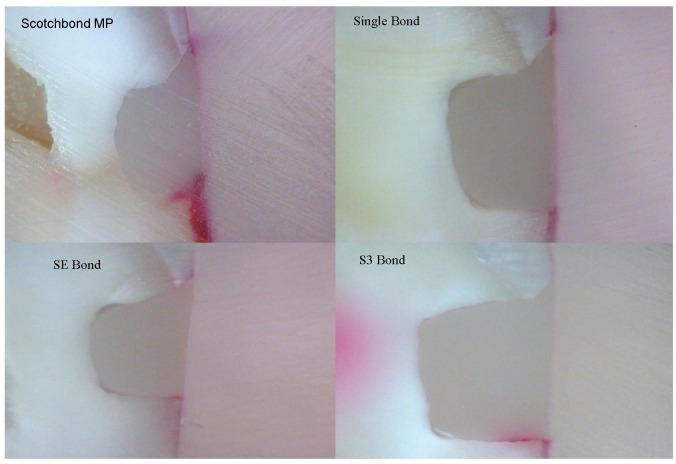
Representation of dye penetration in groups without hydrophobic layer.

Microleakage scores obtained for each group are shown in Table 1. None of the adhesives tested in this study completely eliminated microleakage (Figures 1 & 2). There were no significant differences in microleakage among the eight groups on the occlusal margins (Kruskal-Wallis p=0.1), but significant differences (Kruskal-Wallis p=0.001) were found on the gingival margins, as groups 7 (Clearfil SE Bond plus Margin Bond; Mann-Whitney p=0.001) and 8 (Clearfil S3 Bond plus Margin Bond; Mann-Whitney p=0.001) showed lower dye penetration compared to the other groups, with no statistically significant difference with each other (Mann-Whitney p=0.7). The addition of one layer of hydrophobic adhesive had no significant effect on the reduction of microleakage in Scotchbond MP (Mann-Whitney p=0.1) and Single Bond (Mann-Whitney p=0.7).

**Table 1:** Microleakage ordinal scores obtained for each experimental group (n = 20)

Groups	Dentinal Margin				Enamel Margin			
	0	1	2	3	0	1	2	3
1	6	1	3	10	12	5	3	0
2	3	3	3	10	13	5	1	0
3	2	8	4	5	7	8	4	0
4	6	13	1	0	10	8	2	0
5	5	7	4	3	14	5	0	0
6	1	2	7	10	12	4	4	0
7	16	4	0	0	15	5	0	0
8	14	5	0	0	12	6	1	0

There was no significant difference between enamel and dentinal microleakage in group 4 (Clearfil S3 Bond; Wilcoxon p= 0.4), group 7 (Clearfil SE Bond plus Margin Bond; Wilcoxon p= 0.7), and group 8 (Clearfil S3 Bond plus Margin Bond; Wilcoxon p=0.3).

## Discussion

This study tested the effect of applying an additional hydrophobic layer at the tooth-restoration interface on microleakage after photo polymerizing of the first layer of dentin adhesive systems.

The first hypothesis of the present study was accepted because on enamel, no differences were found among the groups regarding the degree of microleakage. This result is consistent with the findings of previous studies, which demonstrated a high rate of perfect marginal adaptation when self-etching agents have been applied on the enamel margin.^15-18^

Clearfil SE Bond or Clearfil S3 Bond etching primer (10-methacryloyloxydecyl dihydrogen phosphate) could produce a less reflective etching pattern of enamel compared with that created by phosphoric acid, although both of them led to retentive micropores in the enamel surface which resin could effectively penetrate.^19-20^ Other researchers reported higher microleakage values on enamel if phosphoric acid etching was not performed.^11,21^

The second hypothesis of our study was not accepted because significant differences were revealed among groups regarding microleakage in gingival margins. The results of this study showed that the additional hydrophobic layer of unfilled resin significantly improved the sealing ability of tested self-etching adhesives. It has been demonstrated that the diffusion of water could occur rapidly through the hybrid layer to the adhesive/resin composite interface.^4^This phenomenon inhibits polymerization and thereby weakens the adhesive/resin composite interface. The additional layer could extract unpolymerized monomers or oligomers from the hybrid layer zones of a poorly polymerized hydrophilic phase that permits water movement within the hybrid layer of self-etching adhesives.^3^ This layer of resin increases the thickness of the adhesive layer, reduces polymerization stress and may improve stress distribution during testing.^10^

The depth of penetration of self-etch adhesives into subsurface dentin varies depending on the acidity of the self-etch system.^22^ Loguercio et al^22^ claimed that the mild self-etching adhesive “Clearfil SE Bond” forms a thin hybrid layer. The thicker the hybrid layer, the lower the initial gap formation.

Even though this study revealed that the least values of microleakage have been found in groups 7 (Clearfil SE Bond + Margin Bond) and 8 (Clearfil S3 Bond + Margin Bond), the result of this study stating “Clearfil S3 Bond without any hydrophobic layer could significantly decrease the microleakage values” supports a recent study suggesting that the choice of the Clearfil S3 Bond has a significant effect on the improvement of sealing ability compared with Single bond, Prompt L-pop and i-Bond.^23^

An additional hydrophobic layer followed by Scotchbond MP could not significantly improve the dentinal seal compared with that of Scotchbond MP. Silva et al^10^ observed that an additional application of the bonding agent could seal the unpolymerized oxygen inhibited layer, thus enabling it to be adequately polymerized. Gueders et al^11^ claimed that the three-step system “Scotchbond MP” is still the best adhesive and shows minimal leakage. This result is in contrast with the findings of our study.

The present study showed that, without a hydrophobic layer, the microleakage of self-etching adhesives is similar to that of total-etch systems, while self-etching adhesive systems included a hydrophobic layer resulting in significantly reduced microleakage. This is inconsistent with a recent study done by Pushpa and Suresh.^6^ Therefore, the third hypothesis of this study was rejected.

The fourth hypothesis was not accepted as well, because the microleakage values in groups 4 (Clearfil S3 Bond), 7 (Clearfil SE Bond plus Margin Bond) and 8 (Clearfil S3 Bond plus Margin Bond) were similar in occlusal and gingival margins. In one study done by Osorio et al,^15^ the demineralization of human dentin was performed with phosphoric acid EDTA or acidic monomers (Clearfil SE Bond and Xeno V). They showed that collagen degradation was higher with phosphoric acid and EDTA. When dentin was demineralized with Clearfil SE Bond or Xeno V, collagen degradation was reduced by up to 30%, and therefore, Clearfil SE Bond could prevent the sealing ability of the gingival margin.^15^ Self-etching primers containing MDP leads to minimal dissolution of smear plugs and limited openings of tubules; this reduces dentin permeability and facilitates penetration, impregnation, and polymerization. MDP also has two hydroxyl groups that may chelate with calcium ions of enamel and dentin.^24^

Because the mechanisms of adhesions are quite different for each product, in the future, a study should be conducted to compare several all-in-one adhesives with and without the application of a hydrophobic layer regarding dentinal microleakage values.

The findings of the present study emphasize the applying an additional hydrophobic layer with self-etch adhesive systems can be improved of sealing ability.
